# A Novel Algebraic Structure of (*α*,*β*)-Complex Fuzzy Subgroups

**DOI:** 10.3390/e23080992

**Published:** 2021-07-30

**Authors:** Hanan Alolaiyan, Halimah A. Alshehri, Muhammad Haris Mateen, Dragan Pamucar, Muhammad Gulzar

**Affiliations:** 1 Department of Mathematics, King Saud University, Riyadh 11451, Saudi Arabia; holayan@ksu.edu.sa; 2 Department of Computer Science and Engineering, King Saud University, Riyadh 11451, Saudi Arabia; Haalshehri@ksu.edu.sa; 3 Department of Mathematics, University of the Punjab, Lahore 54590, Pakistan; harism.math@gmail.com; 4 Department of Logistics, Military Academy, University of Defence in Belgrade, 11000 Belgrade, Serbia; 5 Department of Mathematics, Government College University Faisalabad, Faisalabad 38000, Pakistan; 98kohly@gmail.com

**Keywords:** complex fuzzy set, (*α*,*β*)-complex fuzzy set, (*α*,*β*)-complex fuzzy subgroup, (*α*,*β*)-complex fuzzy normal subgroup

## Abstract

A complex fuzzy set is a vigorous framework to characterize novel machine learning algorithms. This set is more suitable and flexible compared to fuzzy sets, intuitionistic fuzzy sets, and bipolar fuzzy sets. On the aspects of complex fuzzy sets, we initiate the abstraction of (α,β)-complex fuzzy sets and then define $\left(\alpha ,\beta \right)$-complex fuzzy subgroups. Furthermore, we prove that every complex fuzzy subgroup is an (α,β)-complex fuzzy subgroup and define (α,β)-complex fuzzy normal subgroups of given group. We extend this ideology to define (α,β)-complex fuzzy cosets and analyze some of their algebraic characteristics. Furthermore, we prove that (α,β)-complex fuzzy normal subgroup is constant in the conjugate classes of group. We present an alternative conceptualization of (α,β)-complex fuzzy normal subgroup in the sense of the commutator of groups. We establish the (α,β)-complex fuzzy subgroup of the classical quotient group and show that the set of all (α,β)-complex fuzzy cosets of this specific complex fuzzy normal subgroup form a group. Additionally, we expound the index of $\left(\alpha ,\beta \right)$-complex fuzzy subgroups and investigate the (α,β)-complex fuzzification of Lagrange’s theorem analog to Lagrange’ theorem of classical group theory.

## 1. Introduction

In 1965, Zadeh [1] presented the theory of fuzzy sets and discussed their initiatory results. The action of fuzzy set theory is a decisive structure to deal vagueness and uncertainty in real life problems. Thus, crisp sets commonly do not have suitable response and feedback for actual worldly conditions of happening issues. In addition, this particular set plays a remarkable role in various scientific fields with wide applications in topological spaces, medical diagnosis determination, coding theory, computer sciences, and module theory.

The idea of fuzzy subgroups was introduced by Rosenfeld [2] in 1971. The abstraction of fuzzy subrings was proposed by Liu [3]. Later, these notions were discussed in [4,5,6]. Atanassov [7] initiated the theory of intuitionistic fuzzy sets and established the basic algebraic properties of intuitionistic fuzzy sets. The complex numbers with fuzzy sets were combined by Buckley [8]. Kim [9] presented the idea of the fuzzy order of elements of a group. Ajmal [10] established the fuzzy homomorphism theorems of fuzzy subgroups. He also discussed the fuzzy quotient group and correspondence theorem.

Ray [11] initiated the notion of Cartesian product of fuzzy subgroup. Moreover, the volume and intricacy which exist in the collected information of our daily life are developing rapidly with the phase shift of data. Then, there is regularly existing various sorts of uncertainty in that information is represented with complicated problems in different disciplines, such as biology, economics, social science, computer science, mathematics, and environmental science. With the development of science and technology, the decision making problems are becoming increasingly difficult.

To overcome this drawback, Ramot et al. [12,13] presented the generalized form of fuzzy set by combining a phase term, called a complex fuzzy set. The efficiency of complex fuzzy logic in the respect of membership has a powerful role to deal with concrete problems. It is highly valuable for calculating unevenness, and also it is very useful way to address ambiguous ideas. Despite its efficacy, we have serious problems about physical features on complex membership related function.

Thus, it is highly essential to formulate extra theories of complicated fuzzy set relating intricate set members. This reasoning is a direct version of traditional fuzzy logic, which results in problem related fuzzy reasoning. Thus, it is not favorable for superficial membership function. This set has a very specific role in wide variety of applications in modern commanding systems especially those that forecast periodic events in which a number of variables are interconnected in complex ways and fuzzy operations cannot run it effectively.

The fundamental set theoretic operations of complex fuzzy sets were presented by Zhang et al. [14]. Recently, the possible applications, which explain the novel ideas, including complex fuzzy sets in forecasting issues, solar activity, and time series were investigated by Thirunavukarasu et al. [15]. The complex fuzzy sets have wide applications in decision making, image restoration, and reasoning schemes. Ameri et al. [16] invented the of Engel fuzzy subgroups in 2015. The notion of complex vague soft sets were defined by Selvachandran [17]. Al-Husban and Salleh [18] developed a connection between complex fuzzy sets and group theory in 2016.

Singh et al. [19] discussed the link between complex fuzzy set and metric spaces. In 2016, Thirunavukarasu et al. [20] depicted the abstraction of complex fuzzy graph and find energy of this newly defined graph. In 2017, Alsarahead and Ahmed [21,22,23] presented a new abstraction of complex fuzzy subgroup. They also introduced novel conception complex fuzzy subring and complex fuzzy soft subgroups. These abstractions are completely different from Rosenfeld fuzzy subgroups [2] and Liu fuzzy subring [3]. The parabolic fuzzy subgroups were introduced by Makamba and Murali [24]. Then, certain algebraic properties of Engel fuzzy subgroups were discussed in [25].

Moreover, the Mohamadzahed et al. [26] established the novel concept of nilpotent fuzzy subgroup. The fuzzy homomorphism structures on fuzzy subgroups was discussed by Addis [27]. The algebraic structure between fuzzy sets and normed rings were proposed in [28]. Gulistan et al. [29] presented the notion of (α,β)-complex fuzzy hyper-ideal and investigated many algebraic properties of this phenomena. Liu and Shi [30] presented a novel framework to fuzzification of lattice, which is known as an *M*-hazy lattice. The complex fuzzy sub-algebra commenced in [31].

Currently, the novel environment of complex fuzzy set in decision making problems has been used frequently [32,33,34,35,36,37,38], owing to the existence of complex fuzzy information in several practical situations. Gulzar et al. [39] published a inventive theory of complex fuzzy subfields. The new development about *Q*-complex fuzzy subring was introduced in [40]. For other useful results of fuzzy subgroups not mentioned in the article, readers are referred to [41,42,43,44,45,46,47].

The motivation of the proposed concept is explained as follows: (1) To present a more generalized concept, i.e., (α,β)-complex fuzzy sets. (2) Note that for α=1 and β=2π, our proposed definition can be converted into a classical complex fuzzy set. The purpose of this paper is to present the study of (α,β)-complex fuzzy sets and (α,β)-complex fuzzy subgroups as a powerful extension of complex fuzzy sets and complex fuzzy subgroups.

We organized this article as follows: Section 2 contains the basic notions of complex fuzzy sets (CFSs), complex fuzzy subgroups (CFSGs), and associated results, which are important for our paper. In Section 3, we expound the abstraction of (α,β)-complex fuzzy set (CFS) and (α,β)-complex fuzzy subgroup ((α,β)-CFSG). We prove that every complex fuzzy subgroup (CFSG) is also an (α,β)-complex fuzzy subgroup ((α,β)-CFSG) and discuss fundamental properties of this newly defined CFSG. In Section 4, we explicate the (α,β)-complex fuzzy cosets and (α,β)-complex fuzzy normal subgroups ((α,β)-CFNSGs) and investigate many algebraic characteristics of these specific groups. Moreover, we find the quotient group with respect to (α,β)-complex fuzzy cosets and prove the (α,β)-complex fuzzy quotient group. We initiate the definition of the index of (α,β)-CFSG and develop the (α,β)-complex fuzzification of Lagrange’s Theorem.

## 2. Preliminaries

In this section, we describe the CFSs and CFSGs, and then we discuss the basic operations of complex fuzzy sets.

**Definition** **1**  ([1])**.** *A fuzzy subset is mapping from universe of discourse to [0,1].*

**Definition** **2**  ([12])**.** *A CFS A of nonempty set Z is function from nonempty set to unit disk and is derived as $\theta_{A}:Z\to \left\{z\in C:\left|z\right|\leq 1\right\}.$ The $\theta_{A}\left(p\right)=\mu_{A}\left(p\right)e^{i\varphi_{A}\left(p\right)}$ is membership function of CFS A, where $i=\sqrt{-1}\;$ both $\mu_{A}\left(p\right)$ and $\varphi_{A}\left(p\right)$ are real valued such that $\mu_{A}\left(p\right)\in \left[0,1\right]$ and $\varphi_{A}\left(p\right)\in \left[0,2\pi \right]$, for all p∈Z.*

**Definition** **3**  ([21])**.** *Let A={(p,A(p)):p∈G} be a fuzzy subset. Then, the set*

$$A_{\pi }=\left\{\left(p,\;A_{\pi }\left(p\right)\right):A_{\pi }\left(p\right)=2\pi A\left(p\right),p\in G\right\}$$

*is called a π-fuzzy subset.*

**Definition** **4**  ([21])**.** *A π-fuzzy set $A_{\pi }$ of group G is called a π-fuzzy subgroup of G if*

*1.*  *$A_{\pi }\left(pq\right)\geq \min\left\{A_{\pi }\left(p\right),A_{\pi }\left(q\right)\right\}$, for all p,q∈G,*
*2.*  *$A_{\pi }\left(p^{-1}\right)\geq A_{\pi }\left(p\right)$, for all p∈G.*

**Definition** **5**  ([21])**.** *Let $A=\{\left(p,\mu_{A}\left(p\right)e^{i\varphi_{A}\left(p\right)}\right):p\in G\}$ and $B=\{\left(p,\mu_{B}\left(p\right)e^{i\varphi_{B}\left(p\right)}\right):p\in G\}$ be two CFSs of G. Then,*

*1.*  *A CFS A is homogeneous CFS if *∀*p,q∈G, we have $\mu_{A}\left(p\right)\;\leq \;\mu_{A}\left(q\right)$ if and only if $\varphi_{A}\left(p\right)\;\leq \;\varphi_{A}\left(q\right).$*
*2.*  *A CFS A is homogeneous CFS with B if *∀*p,q∈G, we have $\mu_{A}\left(p\right)\;\leq \;\mu_{B}\left(p\right)$ if and only if $\varphi_{A}\left(p\right)\;\leq \;\varphi_{B}\left(p\right).$*

**Definition** **6**  ([14])**.** *Let $A=\{\left(p,\mu_{A}\left(p\right)e^{i\varphi_{A}\left(p\right)}\right):p\in G\}$ and $B=\{\left(p,\mu_{B}\left(p\right)e^{i\varphi_{B}\left(p\right)}\right):p\in G\}$ be a CFSs of set P. Then, the operation of intersection and union is defined as:*

$$\left(A\cap B\right)\left(p\right)=\mu_{A\cap B}\left(p\right)e^{i\varphi_{A\cap B}\left(p\right)}=\min\left\{\mu_{A}\left(p\right)e^{i\varphi_{A}\left(p\right)},\mu_{B}\left(p\right)e^{i\varphi_{B}\left(p\right)}\right\}\;,\;\forall \;p\in G.$$

$$\left(A\cup B\right)\left(p\right)=\mu_{A\cup B}\left(p\right)e^{i\varphi_{A\cup B}\left(p\right)}=\max\left\{\mu_{A}\left(p\right)e^{i\varphi_{A}\left(p\right)},\mu_{B}\left(p\right)e^{i\varphi_{B}\left(p\right)}\right\}\;,\;\forall \;p\in G.$$

**Definition** **7**  ([21])**.** *Let $A=\{\left(p,\mu_{A}\left(p\right)e^{i\varphi_{A}\left(p\right)}\right):p\in G\}$ be a homogeneous CFS of group G. Then, A is called CFSG of group G if the following conditions hold.*

*1.*  $\mu_{A}\left(pq\right)e^{i\varphi_{A}\left(pq\right)}\geq \min\left\{\;\mu_{A}\left(p\right)e^{i\varphi_{A}\left(p\right)},\;\mu_{A}\left(q\right)e^{i\varphi_{A}\left(q\right)}\right\}\;,$
*2.*  *$\mu_{A}\left(p^{-1}\right)e^{i\varphi_{A}\left(p^{-1}\right)}\geq \mu_{A}\left(p\right)e^{i\varphi_{A}\left(p\right)},$ for all p,q∈G.*

**Definition** **8**  ([21])**.** *A homogeneous CFS $A=\{\left(p,\mu_{A}\left(p\right)e^{i\varphi_{A}\left(p\right)}\right):p\in G\}$ of group G is said to be CFNSG of group G if:$\;\mu_{A}\left(pq\right)e^{i\varphi_{A}\left(pq\right)}=\mu_{A}\left(qp\right)e^{i\varphi_{A}\left(qp\right)}$, for all p,q∈G.*

## 3. Algebraic Properties of (α,β)-Complex Fuzzy Subgroups

In this section, we define the hybrid models of (α,β)-CFSs and (α,β)-CFSGs. We prove that every CFSG is also (α,β)-CFSG but the converse may not be true generally, and we discuss some basic characterization of this phenomenon.

**Definition** **9.** *Let $A=\left\{(p,\mu_{A}(p\right)e^{i\varphi_{A}\left(p\right)}):p\in G\}$ be CFS of group G, for any α∈[0,1] and β∈[0,2π], such that $\mu_{A}\left(p\right)\leq \alpha$ and $\varphi_{A}\left(p\right)\leq \beta$ or $(\mu_{A}\left(p\right)\geq \alpha \;\mathrm{and}\;\varphi_{A}\left(p\right)\geq \beta ).$ Then, the set $A_{\left(\alpha ,\beta \right)}$ is called an (α,β)-CFS and is defined as:*

$$\mu_{A_{\alpha }}\left(p\right)e^{i{\varphi_{A}}_{\beta }\left(p\right)}=\min\{\mu_{A}\left(p\right)e^{i{\varphi_{A}}_{\beta }\left(p\right)},\;\alpha e^{i\beta }\}\;=\min\left\{\mu_{A}\left(p\right),\;\alpha \right\}e^{i\min\{\varphi_{A}\left(p\right),\;\beta \}\;},$$

*where $\mu_{A_{\alpha }}\left(p\right)=\min\{\mu_{A}\left(p\right),\;\alpha \}\;$ and $\varphi_{A_{\beta }}\left(p\right)=\min\{\varphi_{A}\left(p\right),\;\beta \}$.*

*In this paper, we shall use $\mu_{A_{\alpha }}\left(p\right)e^{i{\varphi_{A}}_{\beta }\left(p\right)}$ and $\mu_{B_{\alpha }}\left(p\right)e^{i{\varphi_{B}}_{\beta }\left(p\right)}$, as a membership function of (α,β)-CFSs $A_{\left(\alpha ,\beta \right)}$ and $B_{\left(\alpha ,\beta \right)}$, respectively.*

**Definition** **10.** *Let $A_{\left(\alpha ,\beta \right)}$ and $B_{\left(\alpha ,\beta \right)}$ be a two (α,β)-CFSs of G. Then,*

*1.*  *A (α,β)-CFS $A_{\left(\alpha ,\beta \right)}$ is homogeneous (α,β)-CFS if, for all p,q∈G, we have $\mu_{A_{\alpha }}\left(p\right)\leq \mu_{A_{\alpha }}\left(q\right)$ if and only if $\varphi_{A_{\beta }}\left(p\right)\leq \varphi_{A_{\beta }}\left(q\right).$*
*2.*  *A (α,β)-CFS $A_{\left(\alpha ,\beta \right)}$ is homogeneous (α,β)-CFS with $B_{\left(\alpha ,\beta \right)}$ if, for all p,q∈G, we have $\mu_{A_{\alpha }}\left(p\right)\leq \mu_{B_{\alpha }}\left(p\right)$ if and only if $\varphi_{A_{\beta }}\left(p\right)\leq \varphi_{B_{\beta }}\left(p\right).$*

In this article, we take (α,β)-CFS as homogeneous (α,β)-CFS.

**Remark** **1.** *It is an interesting that we obtain classical CFS A by taking the value of α=1 and β=2π in the above definition.*

**Remark** **2.** *Let $M_{\left(\alpha ,\beta \right)}$ and $N_{\left(\alpha ,\beta \right)}$ be two (α,β)-CFSs of group G. Then, ${\left(M\cap N\right)}_{\left(\alpha ,\beta \right)}=M_{\left(\alpha ,\beta \right)}\cap N_{\left(\alpha ,\beta \right)}.$*

**Definition** **11.** *Let $A_{\left(\alpha ,\beta \right)}$ be an (α,β)-CFS of group G for α∈[0,1] and β∈[0,2π]. Then, $A_{\left(\alpha ,\beta \right)}$ is called (α,β)-complex fuzzy subgroupoid of group G if it satisfies the following axiom:*

$$\begin{array}{c} \mu_{A_{\alpha }}\left(pq\right)e^{i\varphi_{A_{\beta }}\left(pq\right)}\geq \min\{\;\mu_{A_{\alpha }}\left(p\right)e^{i\varphi_{A_{\beta }}\left(p\right)},\;\mu_{A_{\alpha }}\left(q\right)e^{i\varphi_{A_{\beta }}\left(q\right)}\}\;. \end{array}$$

**Definition** **12.** *Let $A_{\left(\alpha ,\beta \right)}$ be an (α,β)-CFS of group G for α∈[0,1] and β∈[0,2π]. Then, $A_{\left(\alpha ,\beta \right)}$ is called (α,β)-CFSG of group G if it satisfies the following axioms:*

*1.*  $\mu_{A_{\alpha }}\left(pq\right)e^{i\varphi_{A_{\beta }}\left(pq\right)}\geq \min\{\;\mu_{A_{\alpha }}\left(p\right)e^{i\varphi_{A_{\beta }}\left(p\right)},\;\mu_{A_{\alpha }}\left(q\right)e^{i\varphi_{A_{\beta }}\left(q\right)}\}\;,$
*2.*  *$\mu_{A_{\alpha }}\left(p^{-1}\right)e^{i\varphi_{A_{\beta }}\left(p^{-1}\right)}\geq \mu_{A_{\alpha }}\left(p\right)e^{i\varphi_{A_{\beta }}\left(p\right)}$ for all p,q∈G.*

**Remark** **3.** *If $A_{\left(\alpha ,\beta \right)}$ is (α,β)-CFSG of group G for α∈[0,1]. Then,*

$$\begin{array}{c} \mu_{A_{\alpha }}\left(p^{-1}q\right)e^{i\varphi_{A_{\beta }}\left(p^{-1}q\right)}\geq \min\{\;\mu_{A_{\alpha }}\left(p\right)e^{i\varphi_{A_{\beta }}\left(p\right)},\;\mu_{A_{\alpha }}\left(q\right)e^{i\varphi_{A_{\beta }}\left(q\right)}\}\;. \end{array}$$

**Theorem** **1.** *If $A_{\left(\alpha ,\beta \right)}$ is an (α,β)-CFSG of group G, for all p,q∈G. Then,*

*1.*  $\mu_{A_{\alpha }}\left(q\right)e^{i\varphi_{A_{\beta }}\left(q\right)}\leq \mu_{A_{\alpha }}\left(e\right)e^{i\varphi_{A_{\beta }}\left(e\right)},$
*2.*  $\mu_{A_{\alpha }}\left(pq^{-1}\right)e^{i\varphi_{A_{\beta }}\left(pq^{-1}\right)}=\mu_{A_{\alpha }}\left(e\right)e^{i\varphi_{A_{\beta }}\left(e\right)},\;$

*which implies that $\mu_{A_{\alpha }}\left(p\right)e^{i\varphi_{A_{\beta }}\left(p\right)}=\mu_{A_{\alpha }}\left(q\right)e^{i\varphi_{A_{\beta }}\left(q\right)}.$*

**Proof.**  Obviously. □

**Theorem** **2.** *Let $A_{\left(\alpha ,\beta \right)}$ be an (α,β)-complex fuzzy subgroupoid of a finite group, and then $A_{\left(\alpha ,\beta \right)}$ is (α,β)-CFSG of finite group.*

**Proof.**  Assume that p∈G. Given that *G* is a finite group; therefore, *p* has finite order *n*. $p^{n}=e$, where *e* is the natural element of group *G*. Then, we have $p^{-1}=p^{n-1}.$ Now, we apply the Definition 11 repeatedly. Then, we obtain

$$\begin{array}{ccc} \mu_{A_{\alpha }}\left(p^{-1}\right)e^{i{\varphi_{A}}_{\beta }\left(p^{-1}\right)} & = & \mu_{A_{\alpha }}\left(p^{n-1}\right)e^{i{\varphi_{A}}_{\beta }\left(p^{n-1}\right)}\\ & = & \mu_{A_{\alpha }}\left(p^{n-2}p\right)e^{i{\varphi_{A}}_{\beta }\left(p^{n-2}p\right)}\\ & \geq & \mu_{A_{\alpha }}\left(p\right)e^{i{\varphi_{A}}_{\beta }\left(p\right)}. \end{array}$$

Hence, we proved the claim. □

**Theorem** **3.** *If $A_{\left(\alpha ,\beta \right)}$ is an (α,β)-CFSG of a group G, let p∈G and $\mu_{A_{\alpha }}\left(p\right)e^{i{\varphi_{A}}_{\beta }\left(p\right)}=\mu_{A_{\alpha }}\left(e\right)e^{i{\varphi_{A}}_{\beta }\left(e\right)}$, and then $\mu_{A_{\alpha }}\left(pq\right)e^{i{\varphi_{A}}_{\beta }\left(pq\right)}=\mu_{A_{\alpha }}\left(q\right)e^{i{\varphi_{A}}_{\beta }\left(q\right)}$, for all q∈G.*

**Proof.**  Given that $\mu_{A_{\alpha }}\left(p\right)e^{i{\varphi_{A}}_{\beta }\left(p\right)}=\mu_{A_{\alpha }}\left(e\right)e^{i{\varphi_{A}}_{\beta }\left(e\right)}$. Then, from Theorem 2, we have

$$\mu_{A_{\alpha }}\left(q\right)e^{i{\varphi_{A}}_{\beta }\left(q\right)}\leq \mu_{A_{\alpha }}\left(p\right)e^{i{\varphi_{A}}_{\beta }\left(p\right)},\forall \;q\in G.$$

Consider

$$\begin{array}{ccc} \mu_{A_{\alpha }}\left(pq\right)e^{i{\varphi_{A}}_{\beta }\left(pq\right)} & \geq & \min\{\mu_{A_{\alpha }}\left(p\right)e^{i{\varphi_{A}}_{\beta }\left(p\right)},\mu_{A_{\alpha }}\left(q\right)e^{i{\varphi_{A}}_{\beta }\left(q\right)}\} \end{array}$$

(1) $$\begin{array}{ccc} \mu_{A_{\alpha }}\left(pq\right)e^{i{\varphi_{A}}_{\beta }\left(pq\right)} & \geq & \mu_{A_{\alpha }}\left(q\right)e^{i{\varphi_{A}}_{\beta }\left(q\right)}.\;\mathrm{From}\;\mathrm{Theorem}\;2. \end{array}$$

$$\begin{array}{ccc} \;\mathrm{Now},\;\mathrm{assume}\;\mathrm{that}\\ \mu_{A_{\alpha }}\left(q\right)e^{i{\varphi_{A}}_{\beta }\left(q\right)} & = & \mu_{A_{\alpha }}\left(p^{-1}pq\right)e^{i{\varphi_{A}}_{\beta }\left(p^{-1}pq\right)}\\ & \geq & \min\{\mu_{A_{\alpha }}\left(p\right)e^{i{\varphi_{A}}_{\beta }\left(p\right)},\mu_{A_{\alpha }}\left(pq\right)e^{i{\varphi_{A}}_{\beta }\left(pq\right)}\}.\\ \mathrm{Again},\;\mathrm{from}\;\mathrm{Theorem}\;2,\;\mathrm{we}\;\mathrm{have}\\ \min\{\mu_{A_{\alpha }}\left(p\right)e^{i{\varphi_{A}}_{\beta }\left(p\right)},\mu_{A_{\alpha }}\left(pq\right)e^{i{\varphi_{A}}_{\beta }\left(pq\right)}\} & = & \mu_{A_{\alpha }}\left(pq\right)e^{i{\varphi_{A}}_{\beta }\left(pq\right)}.\\ \mathrm{Therefore},\;\mathrm{we}\;\mathrm{obtain} \end{array}$$

(2) $$\begin{array}{ccc} \mu_{A_{\alpha }}\left(q\right)e^{i{\varphi_{A}}_{\beta }\left(q\right)} & \geq & \mu_{A_{\alpha }}\left(pq\right)e^{i{\varphi_{A}}_{\beta }\left(pq\right)},\mathrm{for}\;\mathrm{all}\;q\in G, \end{array}$$

$$\begin{array}{ccc} \mathrm{From}\;\mathrm{Equations}\;(1)\;\mathrm{and}\;(2),\;\mathrm{we}\;\mathrm{have}\\ \mu_{A_{\alpha }}\left(q\right)e^{i{\varphi_{A}}_{\beta }\left(q\right)} & = & \mu_{A_{\alpha }}\left(pq\right)e^{i{\varphi_{A}}_{\beta }\left(pq\right)},\mathrm{for}\;\mathrm{all}\;q\in G. \end{array}$$

This establishes the proof. □

**Theorem** **4.** *Every CFSG of group G is also (α,β)-CFSG of G.*

**Proof.**  Let *A* be CFSG of group *G*, for any p,q∈G. Consider

$$\begin{array}{ccc} \mu_{A_{\alpha }}\left(pq\right)e^{i\varphi_{A_{\beta }}\left(pq\right)} & = & \min\{\mu_{A}\left(pq\right)e^{i\varphi_{A}\left(pq\right)},\;\alpha e^{i\beta }\}\;\\ & \geq & \min\{\min\{\mu_{A}\left(p\right)e^{i\varphi_{A}\left(p\right)},\;\mu_{A}\left(q\right)e^{i\varphi_{A}\left(q\right)}\}\;,\;\alpha e^{i\beta }\}\;\\ & = & \min\{\min\{\mu_{A}\left(p\right)e^{i\varphi_{A}\left(p\right)},\alpha e^{i\beta }\}\;,\min\{\mu_{A}\left(q\right)e^{i\varphi_{A}\left(q\right)},\;\alpha e^{i\beta }\}\;\}\\ & = & \min\{\mu_{A_{\alpha }}\left(p\right)e^{i\varphi_{A_{\beta }}\left(p\right)},\mu_{A_{\alpha }}\left(q\right)e^{i\varphi_{A_{\beta }}\left(q\right)}\}\;. \end{array}$$

Further, we assume that

$$\begin{array}{ccc} \mu_{A_{\alpha }}\left(p^{-1}\right)e^{i\varphi_{A_{\beta }}\left(p^{-1}\right)} & = & \min\{\;\mu_{A}\left(p^{-1}\right)e^{i\varphi_{A}\left(p^{-1}\right)},\alpha e^{i\beta }\;\}\;\\ & \geq & \min\{\;\mu_{A}\left(p\right)e^{i\varphi_{A}\left(p\right)},\alpha e^{i\beta }\;\}\;\\ & = & \mu_{A_{\alpha }}\left(p\right)e^{i\varphi_{A_{\beta }}\left(p\right)}. \end{array}$$

This establishes the proof. □

**Remark** **4.** *If $A_{\left(\alpha ,\beta \right)}$-CFSG then it is not necessary A is CFSG.*

**Example** **1.** *Let G={e,r,s,rs} be the Klein four group. One can see that $A=\;\{<e,\;0.1e^{i\frac{\pi }{9}}>,<s,0.3e^{i\frac{\pi }{3}}>,<r,0.3e^{i\frac{\pi }{3}}>,\;<rs,\;0.2e^{i\frac{\pi }{6}}>\}$ is not CFSG of G. Take α=0.01 and $\beta =\frac{\pi }{10}$ then easily we can see that $\mu_{A}\left(p\right)e^{i\varphi_{A}\left(p\right)}>\alpha e^{i\beta }$, for all y∈G. Then, we obtain $\mu_{A_{\alpha }}\left(p\right)e^{i\varphi_{A_{\beta }}\left(p\right)}=\;\alpha e^{i\beta },\;\forall \;p\in G.$ Therefore, $\mu_{A_{\alpha }}\left(pq\right)e^{i\varphi_{A_{\beta }}\left(pq\right)}\geq \min\left\{\mu_{A_{\alpha }}\left(p\right)e^{i\varphi_{A_{\beta }}\left(p\right)},\;\mu_{A_{\alpha }}\left(q\right)e^{i\varphi_{A_{\beta }}\left(q\right)}\right\}$, for all p,q∈G. Moreover, $r^{-1}=r,\;s^{-1}=s,\;{\left(rs\right)}^{-1}=rs$.*

*Thus, $\mu_{A_{\alpha }}\left(p^{-1}\right)e^{i\varphi_{A_{\beta }}\left(p^{-1}\right)}\geq \;\mu_{A_{\alpha }}\left(p\right)e^{i\varphi_{A_{\beta }}\left(p\right)}$. Hence, $A_{\left(\alpha ,\beta \right)}\;$ is (α,β)-CFSG.*

**Theorem** **5.** *Let A be a CFS of group G such that $\mu_{A}\left(p^{-1}\right)e^{i\varphi_{A}\left(p^{-1}\right)}=\mu_{A}\left(p\right)e^{i\varphi_{A}\left(p\right)},$∀p∈G. Let $\alpha e^{i\beta }\leq re^{i\omega }$ such that α≤r and β≤ω, where $re^{i\omega }=\min\left\{\mu_{A}\left(p\right)e^{i\varphi_{A}\left(p\right)}:p\in G\right\}$ and α,r∈[0,1] and β,ω∈[0,2π]. Then, $A_{\left(\alpha ,\beta \right)}$ is an (α,β)-CFSG of G.*

**Proof.**  Note that $\alpha e^{i\beta }\leq re^{i\omega }$ implies that $\min\left\{\mu_{A}\left(p\right)e^{i\varphi_{A}\left(p\right)}\;:p\in G\right\}\geq \;\alpha e^{i\beta }$, which implies that $\min\left\{\mu_{A}\left(p\right)e^{i\varphi_{A}\left(p\right)},\alpha e^{i\beta }\right\}=\;\alpha e^{i\beta }$, for all p∈G, which implies that $\mu_{A_{\alpha }}\left(p\right)e^{i\varphi_{A_{\beta }}\left(p\right)}=\alpha e^{i\beta }.$

$$\begin{array}{ccc} \mu_{A_{\alpha }}\left(pq\right)e^{i\varphi_{A_{\beta }}\left(pq\right)} & \geq & \min\{\mu_{A_{\alpha }}\left(p\right)e^{i\varphi_{A_{\beta }}\left(q\right)},\;\mu_{A_{\alpha }}\left(q\right)e^{i\varphi_{A_{\beta }}\left(q\right)}\}.\\ \mathrm{Moreover},\;\;\;\;\;\;\;\;\;\;\;\;\;\;\;\mu_{A}\left(p^{-1}\right)e^{i\varphi_{A}\left(p^{-1}\right)} & = & \mu_{A}\left(p\right)e^{i\varphi_{A}\left(p\right)},\forall \;p\in G.\\ \mathrm{This}\;\mathrm{implies}\;\mathrm{that}\;\;\;\;\;\;\;\;\;\mu_{A_{\alpha }}\left(p^{-1}\right)e^{i\varphi_{A_{\beta }}\left(p^{-1}\right)} & = & \mu_{A_{\alpha }}\left(p\right)e^{i\varphi_{A_{\beta }}\left(p\right)}. \end{array}$$

Hence, $A_{\left(\alpha ,\beta \right)}\;$ is (α,β)-CFSG of *G*. □

**Theorem** **6.** *If $M_{\left(\alpha ,\beta \right)}$ and $N_{\left(\alpha ,\beta \right)}$ are two (α,β)-CFSGs of G, then $M_{\left(\alpha ,\beta \right)}\cap N_{\left(\alpha ,\beta \right)}$ is also (α,β)-CFSG of G.*

**Proof.**  Given that $M_{\left(\alpha ,\beta \right)}$ and $N_{\left(\alpha ,\beta \right)}$ are two (α,β)-CFSGs of *G*, for any p,q∈G.

$$\begin{array}{ccc} \mathrm{Consider},\;\;\;\;\;\;\;\;\;\;\;\;\;\;\;\;\;\;\;\;\;\;\;\;\;\;\;\\ \mu_{{\left(M\cap N\right)}_{\alpha }}\left(pq\right)e^{\varphi_{{\left(M\cap N\right)}_{\beta }}\left(pq\right)} & = & \mu_{M_{\alpha }\cap N_{\alpha }}\left(pq\right)e^{i\varphi_{M_{\beta }\cap N_{\beta }}\left(pq\right)}\\ & = & \min\{\mu_{M_{\alpha }}\left(pq\right)e^{i\varphi_{M_{\beta }}\left(pq\right)},\;\mu_{N_{\alpha }}\left(pq\right)e^{i\varphi_{N_{\beta }}\left(pq\right)}\}\\ & \geq & \min\left\{\begin{array}{c} \min\{\mu_{M_{\alpha }}\left(p\right)e^{i\varphi_{M_{\beta }}\left(p\right)},\;\mu_{M_{\alpha }}\left(q\right)e^{i\varphi_{M_{\beta }}\left(q\right)}\}\;,\\ \min\{\mu_{N_{\alpha }}\left(p\;\right)e^{i\varphi_{N_{\beta }}\left(p\right)}\;\mu_{N_{\alpha }}\left(q\right)e^{i\varphi_{N_{\beta }}\left(q\right)}\}\;. \end{array}\right\}\\ & = & \min\left\{\begin{array}{c} \min\{\mu_{M_{\alpha }}\left(p\right)e^{i\varphi_{M_{\beta }}\left(p\right)},\mu_{N_{\alpha }}\left(p\right)e^{i\varphi_{N_{\beta }}\left(p\right)}\}\;,\\ \min\{\mu_{M_{\alpha }}\left(q\right)e^{i\varphi_{M_{\beta }}\left(q\right)},\mu_{N_{\alpha }}\left(q\right)e^{i\varphi_{N_{\beta }}\left(q\right)}\}. \end{array}\right\}\\ & = & \min\{\mu_{M_{\alpha }\cap N_{\alpha }}\left(p\right)e^{i\varphi_{N_{\beta }\cap M_{\beta }}\left(p\right)},\;\mu_{N_{\alpha }\cap M_{\alpha }}\left(q\right)e^{i\varphi_{M_{\beta }\cap N_{\beta }}\left(q\right)}\}\;\\ & = & \min\{\mu_{{\left(M\cap N\right)}_{\alpha }}\left(p\right)e^{i\varphi_{{\left(M\cap N\right)}_{\beta }}\left(p\right)},\mu_{{\left(M\cap N\right)}_{\alpha }}\left(q\right)e^{i\varphi_{{\left(M\cap N\right)}_{\beta }}\left(q\right)}\}\;.\\ \mathrm{Further},\;\;\;\;\;\;\;\;\;\;\;\;\;\;\;\;\;\;\;\;\;\;\;\;\;\\ \mu_{{\left(M\cap N\right)}_{\alpha }}\left(p^{-1}\right)e^{\varphi_{{\left(M\cap N\right)}_{\beta }}\left(p^{-1}\right)} & = & \mu_{M_{\alpha }\cap N_{\alpha }}\left(p^{-1}\right)e^{i\varphi_{M_{\beta }\cap N_{\beta }}\left(p^{-1}\right)}\\ & = & \min\{\mu_{M_{\alpha }}\left(p^{-1}\right)e^{i\varphi_{M_{\beta }}\left(p^{-1}\right)},\;\mu_{N_{\alpha }}\left(p^{-1}\right)e^{i\varphi_{N_{\beta }}\left(p^{-1}\right)}\}\\ & \geq & \min\{\mu_{M_{\alpha }}\left(p\right)e^{i\varphi_{M_{\beta }}\left(p\right)},\;\mu_{N_{\alpha }}\left(p\right)e^{i\varphi_{N_{\beta }}\left(p\right)}\}\\ & = & \mu_{{\left(M\cap N\right)}_{\alpha }}\left(p\right)e^{\varphi_{{\left(M\cap N\right)}_{\beta }}\left(p\right)}. \end{array}$$

Consequently, $M_{\left(\alpha ,\beta \right)}\cap N_{\left(\alpha ,\beta \right)}$ is (α,β)-CFSG of *G*. □

**Remark** **5.** *The union of two (α,β)-CFSGs may not be (α,β)-CFSG.*

**Example** **2.** *Consider a symmetric group $S_{4}$ of all permutation of four elements. Define two (α,β)-CFSGs $A_{\left(0.6,\pi \right)}$ and $B_{\left(0.6,\pi \right)}$ of $S_{4}$ for the value $\alpha e^{i\beta }=0.6e^{\pi }$ given as:*

$$A_{\left(0.6,\pi \right)}\left(p\right)=\left\{\begin{array}{l} 0.5e^{\pi /2},\;\;\mathrm{if}\;p\in <\left(1\;3\right)>\\ 0.4e^{\pi /4},\;\;\mathrm{otherwise}\;\;\;\;\;\;\;\;\; \end{array}\right.\mathrm{and}\;B_{\left(0.6,\pi \right)}\left(p\right)=\left\{\begin{array}{l} 0.6e^{\pi },\;\;\mathrm{if}\;p\in <\left(1\;3\;2\;4\right)>\\ 0.3e^{\pi /5},\;\;\mathrm{otherwise}\;\;\;\;\;\;\;\;\;\;\;\;\;\; \end{array}\right.$$

*This implies that*

$$\left(A_{\left(0.6,\pi \right)}\cup B_{\left(0.6,\pi \right)}\right)\left(p\right)=\left\{\begin{array}{c} 0.6e^{\pi },\;\;\mathrm{if}\;p\in <\left(1\;32\;4\right)>\;\;\;\;\;\;\;\;\;\;\;\;\;\;\;\;\;\;\;\;\;\\ 0.5e^{\pi /2},\;\;\mathrm{if}\;p\in <\left(1\;3\right)>-<\left(1\;3\;2\;4\right)>\\ 0.4e^{\pi /4},\;\;\mathrm{otherwise}\;\;\;\;\;\;\;\;\;\;\;\;\;\;\;\;\;\;\;\;\;\;\;\;\;\;\;\;\; \end{array}\right.$$

*Take p=(12)(34), q=(13) and pq=(1234). Therefore, $\left(A_{\left(0.6,\pi \right)}\cup B_{\left(0.6,\pi \right)}\right)\left(p\right)=0.6e^{\pi }.$ $\left(A_{\left(0.6,\pi \right)}\cup B_{\left(0.6,\pi \right)}\right)\left(q\right)=0.5e^{\pi /2}$ and $\left(A_{\left(0.6,\pi \right)}\cup B_{\left(0.6,\pi \right)}\right)\left(pq\right)=0.4e^{\pi /4}.$*

*Clearly, we can see that*

$$\left(A_{\left(0.6,\pi \right)}\cup B_{\left(0.6,\pi \right)}\right)\left(pq\right)≱\min\left\{\left(A_{\left(0.6,\pi \right)}\cup B_{\left(0.6,\pi \right)}\right)\left(p\right),\left(A_{\left(0.6,\pi \right)}\cup B_{\left(0.6,\pi \right)}\right)\left(y\right)\right\}$$

*Hence, this proves the claim.*

## 4. (α,β)-Complex Fuzzification of Lagrange’s Theorem

In this section, we investigate the algebraic attributions of (α,β)-CFNSGs. We start the study of the concept of (α,β)-complex fuzzy cosets of (α,β)-CFSG and develop a quotient group induced by this particular CFNSGs. We also establish (α,β)-CFSG of classical quotient group and prove some important properties of these CFNSGs. Moreover, we discuss (α,β)-complex fuzzification of Lagrange’s heorem.

**Definition** **13.** *Let $A_{\left(\alpha ,\beta \right)}$ be an (α,β)-CFSG of group G, where α∈[0,1] and β∈[0,2π]. Then, the (α,β)-CFS ${pA}_{\left(\alpha ,\beta \right)}\left(x\right)=\left\{\left(x,\mu_{{pA}_{\alpha }}\left(x\right)e^{{i\varphi }_{{{pA}_{\beta }}^{\left(x\right)}}}\right),\;x\in G\right\}$ of G is called a (α,β)-complex fuzzy left coset of G determined by $A_{\left(\alpha ,\beta \right)}$ and p and is described as: *

$$\mu_{{pA}_{\alpha }}\left(x\right)e^{{i\varphi }_{pA_{\beta }}\left(x\right)}=\mu_{A_{\alpha }}\left(p^{-1}x\right)e^{{i\varphi }_{A_{\beta }}\left(p^{-1}x\right)}=\min\{\mu_{A}\left(p^{-1}x\right)e^{i\varphi_{A}\left(p^{-1}x\right)},\;\alpha e^{i\beta }\}\;,\mathrm{for}\;\mathrm{all}\;x,p\in G.$$

*Similarly we can define (α,β)-complex fuzzy right coset $A_{\left(\alpha ,\beta \right)}p\left(x\right)=\left\{\left(x,\mu_{A_{\alpha }p}\left(x\right)e^{{i\varphi }_{A_{\beta }p}\left(x\right)}\right),\right\}$x∈G of of G determined by $A_{\left(\alpha ,\beta \right)}$ and p and is described as:*

$$\;\mu_{A_{\alpha x)e^{{i\varphi }_{A_{\beta }p}\left(x\right)}}}=\mu_{A_{\alpha }}\left(xp^{-1}\right)e^{{i\varphi }_{A_{\beta }}\left(xp^{-1}\right)}=\min\{\mu_{A}\left(xp^{-1}\right)e^{i\varphi_{A}\left(xp^{-1}\right)},\;\alpha e^{i\beta }\}\;,\mathrm{for}\;\mathrm{all}\;x\;,\;p\in G.$$

The following example illustrates the notion of (α,β)-complex fuzzy cosets of $A_{\left(\alpha ,\beta \right)}$.

**Example** **3.** *Take G={(1),(24),(13),(12)(34),(13)(24),(14)(23),(1234),(1432)} a permutation group of order 8. Define (α,β)-CFSG of G for the value of α=0.6 and β=π/2 as follows:*

$$A_{\left(0.6,\pi /2\right)}\left(x\right)=\;\left\{\begin{array}{c} 0.6e^{\pi /2}\;\mathrm{if}\;x\in \left\{\left(1\right),\left(1\;3\right)\left(2\;4\right)\right\},\\ 0.5e^{\pi /4},\;\mathrm{if}\;x\in \left\{\left(1\;4\right)\left(2\;3\right),\left(1\;2\right)\left(3\;4\right)\right\},\\ 0.4e^{\pi /6},\;\mathrm{otherwise} \end{array}\right.$$

*From Definition 13, we have $\mu_{pA_{\left(0.6,\pi /2\right)}}\left(x\right)e^{\varphi_{pA_{\left(0.6,\pi /2\right)}}\left(x\right)}=\mu_{A_{\left(0.6,\pi /2\right)}}\left(p^{-1}x\right)e^{\varphi_{A_{\left(0.6,\pi /2\right)}}\left(p^{-1}x\right)}.$*

*Hence, the (0.6,π/2)-complex fuzzy left coset of $A_{\left(0.6,\pi /2\right)}\left(x\right)$ in G for p=(24) is as follows:*

$$pA_{\left(0.6,\pi /2\right)}\left(x\right)=\;\left\{\begin{array}{c} 0.6e^{\pi /2}\;\mathrm{if}\;x\in \left\{\left(1\;3\right),\left(2\;4\right)\right\},\\ 0.5e^{\pi /4},\;\mathrm{if}\;x\in \left\{\left(1\;4\;3\;2\right),\left(1\;2\;3\;4\right)\right\},\\ 0.4e^{\pi /6},\;\mathrm{otherwise} \end{array}\right.$$

*Similarly, one can find (0.6,π/2)-complex fuzzy left coset of $A_{\left(0.6,\pi /2\right)}\left(x\right)$, for each p∈G.*

**Definition** **14.** *Let $A_{\left(\alpha ,\beta \right)}$ be an (α,β)-CFSG of group G, where α∈[0,1] and β∈[0,2π]. Then, $A_{\left(\alpha ,\beta \right)}$ is called a (α,β)-CFNSG if $A_{\left(\alpha ,\beta \right)}\left(pq\right)=A_{\left(\alpha ,\beta \right)}\left(qp\right).$ Equivalently (α,β)-CFSG $A_{\left(\alpha ,\beta \right)}$ is (α,β)-CFNSG of group G if: $A_{\left(\alpha ,\beta \right)}p\left(q\right)=pA_{\left(\alpha ,\beta \right)}\left(q\right)$, for all p,q∈G.*

Note that (1,2π)-CFNSG is classical CFNSG of *G*.

**Remark** **6.** *Let $A_{\left(\alpha ,\beta \right)}$ be an (α,β)-CFNSG of group G. Then, $A_{\left(\alpha ,\beta \right)}\left(q^{-1}pq\right)=A_{\left(\alpha ,\beta \right)}\left(p\right),$ for all p,q∈G.*

**Theorem** **7.** *If A is CFNSG of group G, then $A_{\left(\alpha ,\beta \right)}$ is an (α,β)-CFNSG of G.*

**Proof.**  Let x,p be any elements of *G*. Therefore, we have

$$\begin{array}{ccc} \mu_{A}\left(p^{-1}x\right)e^{i\varphi_{A}\left(p^{-1}x\right)} & = & \mu_{A}\left({xp}^{-1}\right)e^{i\varphi_{A}\left(xp^{-1}\right)}.\\ \mathrm{This}\;\mathrm{implies}\;\mathrm{that}\;\{\mu_{A}\left(p^{-1}x\right)e^{i\varphi_{A}\left(p^{-1}x\right)},\alpha e^{i\beta }\} & = & \min\{\mu_{A}\left({xp}^{-1}\right)e^{i\varphi_{A}\left(xp^{-1}\right)}{,\alpha e}^{i\beta }\}\;,\\ \mathrm{which}\;\mathrm{implies}\;\mathrm{that}\;\mu_{{pA}_{\alpha }}\left(x\right)e^{{i\varphi }_{{pA}_{\beta }}\left(x\right)} & = & \mu_{A_{\alpha }p}\left(x\right)e^{{i\varphi }_{A_{\beta }p}\left(x\right)}.\\ \mathrm{This}\;\mathrm{implies}\;\mathrm{that}\;{pA}_{\left(\alpha ,\beta \right)}\left(x\right) & = & A_{\left(\alpha ,\beta \right)}p\left(x\right). \end{array}$$

Consequently, $A_{\left(\alpha ,\beta \right)}$ is (α,β)-CFNSG of G. □

The converse of the above result does not hold generally. In the following example, we explain this fact.

**Example** **4.** *Let $G=D_{3\;}=<r,s:\;r^{3}=s^{2}=e,\;sr=r^{2}s>$ be the dihedral group.*

*Let A be a CFS of G and described as:*

$$A=\;\left\{\begin{array}{c} 0.8e^{\pi /3}\;if\;x\in <s>,\\ 0.7e^{\pi /6},\;\mathrm{otherwise} \end{array}\right.$$

*Note that A is not a CFNSG of group G. For ${\mu_{A}\left(r^{2}\left(rs\right)\right)e^{i\varphi_{A}\left(r^{2}\left(rs\right)\right)}=\;\;\;0.8e\;}^{\pi /3\;}\neq \;{0.7e}^{\pi /6}=\mu_{A}\left(\left(rs\right)r^{2}\right)e^{i\varphi_{A}\left(\left(rs\right)r^{2}\right)}$. Now, we take ${\alpha e}^{i\beta }={0.5e}^{i^{\pi /9}}$, and we obtain*

$$\mu_{pA_{0.5}}\left(x\right)e^{i\varphi_{pA_{\pi /9}}\left(x\right)}=\min\left\{\mu_{A}\left(p^{-1}x\right)e^{i\varphi_{A}\left(p^{-1}x\right)},0.5e^{i\pi /9}\right\}=0.5e^{\frac{i\pi }{9}}$$

$$=\;\min\{\mu_{A}\left(xp^{-1}\right)e^{{i\varphi }_{A}{(xp}^{-1})},0.5e^{i\pi /9}\}\;=\mu_{A_{0.5}p}\left(x\right)e^{i\varphi_{A_{\pi /9}p}\left(x\right)}.$$

Next, we prove that every (α,β)-CFSG of group *G* will be (α,β)-CFNSG of group *G*, for some specific values of α and β. In this direction, we prove the following results.

**Theorem** **8.** *Let $A_{\left(\alpha ,\beta \right)}$ be (α,β)-CFSG of group G such that ${\alpha e}^{i\beta }<{re}^{i\omega \;}$ such that α≤r and β≤ω, where $\;{re}^{i\omega }=\min\{\mu_{A}\left(x\right)e^{i\varphi_{A}\left(x\right)},\forall \;x\in G\;\}$ and α,r∈[0,1] and β,ω∈[0,2π]. Then, $A_{\left(\alpha ,\beta \right)}$ is a (α,β)-complex fuzzy normal subgroup of group G.*

**Proof.**  Given that ${\alpha e}^{i\beta }\leq {re}^{i\omega \;}$ implies that $\min\{\mu_{A}\left(x\right)e^{i\varphi_{A}\left(x\right)}:\;\mathrm{for}\;\mathrm{all}\;x\in G\}\;\geq {\alpha e}^{i\beta },$ which implies that $\mu_{A}\left(x\right)e^{i\varphi_{A}\left(x\right)}\geq {\alpha e}^{i\beta }$, forallx∈G.

Thus, $\mu_{{pA}_{\alpha }}\left(x\right)e^{{i\varphi }_{{pA}_{\beta }}\left(x\right)}=\min\left\{\mu_{A}\left(p^{-1}x\right)e^{i\varphi_{A}\left(p^{-1}x\right)},\alpha e^{i\beta }\right\}={\alpha e}^{i\beta },$ for any x∈G.

Similarly, $\mu_{A_{\alpha }p}\left(x\right)e^{{i\varphi }_{A_{\beta }p}\left(x\right)}=\min\{\mu_{A}\left(xp^{-1}\right)e^{i\varphi_{A}\left(xp^{-1}\right)},\;{\alpha e}^{i\beta }\}\;={\alpha e}^{i\beta }.$

This implies that $\mu_{{pA}_{\alpha }}\left(x\right)e^{{i\varphi }_{{pA}_{\beta }}\left(x\right)}=\mu_{A_{\alpha }p}\left(x\right)e^{{i\varphi }_{A_{\beta }p}\left(x\right)}.$ Hence, we proved the result. □

**Theorem** **9.** *Let $A_{\left(\alpha ,\beta \right)}$ be an (α,β)-CFSG of a group G, then $A_{\left(\alpha ,\beta \right)}$ is an (α,β)-complex fuzzy normal subgroup if and only if $A_{\left(\alpha ,\beta \right)}$ is constant in the in the conjugacy class of group G.*

**Proof.**  Assume that $A_{\left(\alpha ,\beta \right)}$ is an (α,β)-complex fuzzy normal subgroup of group *G*. Then, we have

$$\begin{array}{ccc} \mu_{A_{\alpha }}\left(q^{-1}pq\right)e^{i{\varphi_{A}}_{\beta }\left(q^{-1}pq\right)} & = & \mu_{A_{\alpha }}\left(pqq^{-1}\right)e^{i{\varphi_{A}}_{\beta }\left(pqq^{-1}\right)}.\\ & = & \mu_{A_{\alpha }}\left(p\right)e^{i{\varphi_{A}}_{\beta }\left(p\right)},\;\forall \;p,q\in G. \end{array}$$

Conversely, suppose that $A_{\left(\alpha ,\beta \right)}$ is constant in all conjugate classes of group *G*. Then,

$$\begin{array}{ccc} \mu_{A_{\alpha }}\left(pq\right)e^{i{\varphi_{A}}_{\beta }\left(pq\right)} & = & \mu_{A_{\alpha }}\left(pqpp^{-1}\right)e^{i{\varphi_{A}}_{\beta }\left(pqpp^{-1}\right)}.\\ & = & \mu_{A_{\alpha }}\left(p\left(qp\right)p^{-1}\right)e^{i{\varphi_{A}}_{\beta }\left(p\left(qp\right)p^{-1}\right)}.\\ & = & \mu_{A_{\alpha }}\left(qp\right)e^{i{\varphi_{A}}_{\beta }\left(qp\right)},\;\forall \;p,q\in G. \end{array}$$

Hence, we prove the claim. □

**Theorem** **10.** *If $A_{\left(\alpha ,\beta \right)}$ is an (α,β)-CFSG of a group G, then $A_{\left(\alpha ,\beta \right)}$ is an (α,β)-complex fuzzy normal subgroup if and only if $\mu_{A_{\alpha }}\left(\left[p,q\right]\right)e^{i{\varphi_{A}}_{\beta }\left(\left[p,q\right]\right)}\geq \mu_{A_{\alpha }}\left(p\right)e^{i{\varphi_{A}}_{\beta }\left(p\right)},\;\forall \;p,q\in G.$*

**Proof.**  Suppose that $A_{\left(\alpha ,\beta \right)}$ is an (α,β)-complex fuzzy normal subgroup of group *G*. Let x,y∈G be element of group. Consider

$$\begin{array}{ccc} \mu_{A_{\alpha }}\left(p^{-1}q^{-1}pq\right)e^{i{\varphi_{A}}_{\beta }\left(p^{-1}q^{-1}pq\right)} & \geq & \min\{\mu_{A_{\alpha }}\left(q^{-1}pq\right)e^{i{\varphi_{A}}_{\beta }\left(q^{-1}pq\right)},\mu_{A_{\alpha }}\left(p^{-1}\right)e^{i{\varphi_{A}}_{\beta }\left(p^{-1}\right)}\}\\ & = & \min\{\mu_{A_{\alpha }}\left(p\right)e^{i{\varphi_{A}}_{\beta }\left(p\right)},\mu_{A_{\alpha }}\left(p\right)e^{i{\varphi_{A}}_{\beta }\left(p\right)}\}\\ \mu_{A_{\alpha }}\left(\left[p,q\right]\right)e^{i{\varphi_{A}}_{\beta }\left(\left[p,q\right]\right)} & \geq & \mu_{A_{\alpha }}\left(p\right)e^{i{\varphi_{A}}_{\beta }\left(p\right)}. \end{array}$$

Conversely, suppose that $\mu_{A_{\alpha }}\left(\left[p,q\right]\right)e^{i{\varphi_{A}}_{\beta }\left(\left[p,q\right]\right)}\geq \mu_{A_{\alpha }}\left(p\right)e^{i{\varphi_{A}}_{\beta }\left(p\right)}.$ Let p,r∈G be an element.

(3) $$\begin{array}{ccc} \mathrm{Consider}\;\mu_{A_{\alpha }}\left(p^{-1}rp\right)e^{i{\varphi_{A}}_{\beta }\left(p^{-1}rp\right)} & = & \mu_{A_{\alpha }}\left(rr^{-1}p^{-1}rp\right)e^{i{\varphi_{A}}_{\beta }\left(rr^{-1}p^{-1}rp\right)}\\ & \geq & \min\{\mu_{A_{\alpha }}\left(r\right)e^{i{\varphi_{A}}_{\beta }\left(r\right)},\mu_{A_{\alpha }}\left(\left[r,p\right]\right)e^{i{\varphi_{A}}_{\beta }\left(\left[r,p\right]\right)}\}\\ & = & \mu_{A_{\alpha }}\left(r\right)e^{i{\varphi_{A}}_{\beta }\left(r\right)} \end{array}$$

(4) $$\begin{array}{ccc} \mathrm{Thus}\;,\mu_{A_{\alpha }}\left(p^{-1}rp\right)e^{i{\varphi_{A}}_{\beta }\left(p^{-1}rp\right)} & \geq & \mu_{A_{\alpha }}\left(r\right)e^{i{\varphi_{A}}_{\beta }\left(r\right)},\;\forall \;r,\;p\in G. \end{array}$$

(5) $$\begin{array}{ccc} \mathrm{Now},\;\mu_{A_{\alpha }}\left(r\right)e^{i{\varphi_{A}}_{\beta }\left(r\right)} & = & \mu_{A_{\alpha }}\left(pp^{-1}rpp^{-1}\right)e^{i{\varphi_{A}}_{\beta }\left(pp^{-1}rpp^{-1}\right)}\\ & \geq & \min\{\mu_{A_{\alpha }}\left(p\right)e^{i{\varphi_{A}}_{\beta }\left(p\right)},\mu_{A_{\alpha }}\left(p^{-1}rp\right)e^{i{\varphi_{A}}_{\beta }\left(p^{-1}rp\right)}\}. \end{array}$$

Now, we expound two possible cases.

**Case** **1** $$\begin{array}{ccc} \mathrm{If}\;\min\{\mu_{A_{\alpha }}\left(p\right)e^{i{\varphi_{A}}_{\beta }\left(p\right)},\mu_{A_{\alpha }}\left(p^{-1}rp\right)e^{i{\varphi_{A}}_{\beta }\left(p^{-1}rp\right)}\} & = & \mu_{A_{\alpha }}\left(p\right)e^{i{\varphi_{A}}_{\beta }\left(p\right)}.\\ \mathrm{Then},\;\mathrm{we}\;\mathrm{obtain}\;\;\mu_{A_{\alpha }}\left(r\right)e^{i{\varphi_{A}}_{\beta }\left(r\right)} & \geq & \mu_{A_{\alpha }}\left(p\right)e^{i{\varphi_{A}}_{\beta }\left(p\right)},\;\forall \;r,\;p\in G. \end{array}$$

This implies that $A_{\left(\alpha ,\beta \right)}$ is a constant mapping and, in this case, the result holds obviously.

**Case** **2** (6) $$\begin{array}{ccc} \mathrm{If}\;\min\{\mu_{A_{\alpha }}\left(p\right)e^{i{\varphi_{A}}_{\beta }\left(p\right)},\mu_{A_{\alpha }}\left(p^{-1}rp\right)e^{i{\varphi_{A}}_{\beta }\left(p^{-1}rp\right)}\} & = & \mu_{A_{\alpha }}\left(p^{-1}rp\right)e^{i{\varphi_{A}}_{\beta }\left(p^{-1}rp\right)}.\\ \mathrm{Then},\;\mathrm{from}\;\mathrm{Equation}\;(5)\;\mathrm{we}\;\mathrm{have}\;\\ \mu_{A_{\alpha }}\left(r\right)e^{i{\varphi_{A}}_{\beta }\left(r\right)}\} & \geq & \mu_{A_{\alpha }}\left(p^{-1}rp\right)e^{i{\varphi_{A}}_{\beta }\left(p^{-1}rp\right)}.\\ \mathrm{In}\;\mathrm{the}\;\mathrm{view}\;\mathrm{of}\;\mathrm{Equations}\;(4)\;\mathrm{and}\;(6)\;\mathrm{we}\;\mathrm{have}\;\\ \mu_{A_{\alpha }}\left(r\right)e^{i{\varphi_{A}}_{\beta }\left(r\right)}\} & = & \mu_{A_{\alpha }}\left(p^{-1}rp\right)e^{i{\varphi_{A}}_{\beta }\left(p^{-1}rp\right)}. \end{array}$$

Hence, $A_{\left(\alpha ,\beta \right)}$ is constant. □

**Theorem** **11.** *Let $\;A_{\left(\alpha ,\beta \right)}$ be (α,β)-CFNSG of group G. Then, the set $A_{\left(\alpha ,\beta \right)}^{e}=\left\{\;x\in G:\;A_{\left(\alpha ,\beta \right)}\left(x^{-1}\right)=A_{\left(\alpha ,\beta \right)}\left(e\right)\;\right\}$* *is a normal subgroup of group G.*

**Proof.**  We know that $A_{\left(\alpha ,\beta \right)}^{e}$≠φ because e∈G. Let $x,\;y\in A_{\left(\alpha ,\beta \right)}^{e}$ be any elements.

Consider

$$\mu_{A_{\alpha }}\left(xy\right)e^{i\varphi_{A_{\beta }}\left(xy\right)}\geq \min\{\mu_{A_{\alpha }}\left(x\right)e^{i\varphi_{A_{\beta }}\left(x\right)},\mu_{A_{\alpha }}\left(y\right)e^{i\varphi_{A_{\beta }}\left(y\right)}\;\}\;=\min\{\mu_{A_{\alpha }}\left(e\right)e^{i\varphi_{A_{\beta }}\left(e\right)},\mu_{A_{\alpha }}\left(e\right)e^{i\varphi_{A_{\beta }}\left(e\right)}\}\;.$$

This implies that $\mu_{A_{\alpha }}\left(xy\right)e^{i\varphi_{A_{\beta }}\left(xy\right)}\geq \mu_{A_{\alpha }}\left(e\right)e^{i\varphi_{A_{\beta }}\left(e\right)}$. However, $\mu_{A_{\alpha }}\left(xy\right)e^{i\varphi_{A_{\beta }}\left(xy\right)}\leq$$\mu_{A_{\alpha }}\left(e\right)e^{i\varphi_{A_{\beta }}\left(e\right)}.$ Therefore, $\mu_{A_{\alpha }}\left(xy\right)e^{i\varphi_{A_{\beta }}\left(xy\right)}=\;\mu_{A_{\alpha }}\left(e\right)e^{i\varphi_{A_{\beta }}\left(e\right)}.$ This implies that $A_{\left(\alpha ,\beta \right)}\left(x^{-1}\right)=A_{\left(\alpha ,\beta \right)}\left(e\right),$ which implies that $xy\in A_{\left(\alpha ,\beta \right)}^{e}$. Further, $\mu_{A_{\alpha }}\left(y^{-1}\right)e^{i\varphi_{A_{\beta }}\left(y^{-1}\right)}\geq \mu_{A_{\alpha }}\left(y\right)e^{i\varphi_{A_{\beta }}\left(y\right)}=\mu_{A_{\alpha }}\left(e\right)e^{i\varphi_{A_{\beta }}\left(e\right)}.$ However, $\mu_{A_{\alpha }}\left(x\right)e^{i\varphi_{A_{\beta }}\left(x\right)}\leq \mu_{A_{\alpha }}\left(e\right)e^{i\varphi_{A_{\beta }}\left(e\right)}$. Thus, $A_{\left(\alpha ,\beta \right)}^{e}$ is subgroup of group *G*. Moreover, let $x\in A_{\left(\alpha ,\beta \right)}^{e}$ and y∈G. We have $\mu_{A_{\left(\alpha ,\beta \right)}}\left(y^{-1}xy\right)e^{i\varphi_{A_{\left(\alpha ,\beta \right)}}\left(y^{-1}xy\right)}=\mu_{A_{\left(\alpha ,\beta \right)}}\left(x\right)e^{i\varphi_{A_{\left(\alpha ,\beta \right)}}\left(x\right)}$. This implies that $y^{-1}xy\in A_{\left(\alpha ,\beta \right)}^{e}$. Hence, $A_{\left(\alpha ,\beta \right)}^{e}$ is a normal subgroup. □

**Theorem** **12.** *Let $A_{\left(\alpha ,\beta \right)}$ be an (α,β)-CFNSG of group G. Then,*

*1.*  ${pA}_{\left(\alpha ,\beta \right)}=qA_{\left(\alpha ,\beta \right)}\;\;\;\;\;\mathrm{if}\;\mathrm{and}\;\mathrm{if}\;\mathrm{only}\;\;p^{-1}q\in A_{\left(\alpha ,\beta \right)}^{e}$
*2.*  $A_{\left(\alpha ,\beta \right)}p\;=A_{\left(\alpha ,\beta \right)}\;q\;\;\;\;\mathrm{if}\;\mathrm{and}\;\mathrm{if}\;\mathrm{only}\;\;{pq}^{-1}\;\in A_{\left(\alpha ,\beta \right)}^{e}$

**Proof.**  For any p,q∈G, we have ${pA}_{\left(\alpha ,\beta \right)}=qA_{\left(\alpha ,\beta \right)}$.

Consider,

$$\begin{array}{ccc} \mu_{A_{\alpha }}\left(p^{-1}q\right)e^{i\varphi_{A_{\beta }}\left(p^{-1}q\right)} & = & \min\{\mu_{A}\left(p^{-1}q\right)e^{i\varphi_{A}\left(p^{-1}q\right)},{\alpha e}^{i\beta }\}\;\\ & = & \min\{\mu_{pA}\left(q\right)e^{i\varphi_{pA}\left(q\right)},{\alpha e}^{i\beta }\}\;\\ & = & \mu_{pA_{\alpha }}\left(q\right)e^{i\varphi_{{pA}_{\beta }}\left(q\right)}\\ & = & \mu_{qA_{\alpha }}\left(q\right)e^{i\varphi_{{qA}_{\beta }}\left(q\right)}\\ & = & \min\{\mu_{A}\left(q^{-1}q\right)e^{i\varphi_{\beta }\left(q^{-1}q\right)},{\alpha e}^{i\beta }\}\\ & = & \min\{\mu_{A}\left(e\right)e^{i\varphi_{A}\left(e\right)},{\alpha e}^{i\beta }\}\\ & = & \;\mu_{A_{\alpha }}\left(e\right)e^{i\varphi_{A_{\beta }}\left(e\right)}. \end{array}$$

Therefore, $p^{-\;1}q\in A_{\left(\alpha ,\beta \right)}^{e}$.

Conversely, let $p^{-1}q\in A_{\left(\alpha ,\beta \right)}^{e}$ implies that $\mu_{A_{\alpha }}\left(p^{-1}q\right)e^{i\varphi_{A_{\beta }}\left(p^{-1}q\right)}=\mu_{A_{\alpha }}\left(e\right)e^{i\varphi_{A_{\beta }}\left(e\right)}.$

$$\begin{array}{ccc} \mathrm{Consider},\;\mu_{{pA}_{\alpha }}\left(a\right)e^{i\varphi_{{pA}_{\beta }}\left(a\right)} & = & \min\{\mu_{A}\left(p^{-1}a\right)e^{i\varphi_{A}\left(p^{-1}a\right)},\alpha e^{i\beta }\}\;\\ & = & \mu_{A_{\alpha }}\left(p^{-1}a\right)e^{i\varphi_{A}\left(p^{-1}a\right)}\\ & = & \mu_{A_{\alpha }}\left(p^{-1}q\right)\left(q^{-1}a\right)e^{i\varphi_{A_{\beta }}\left(p^{-1}q\right)\left(q^{-1}a\right)}\\ & \geq & \min\{\mu_{A_{\alpha }}\left(p^{-1}q\right)e^{i\varphi_{A_{\beta }}\left(p^{-1}q\right)},\mu_{A_{\alpha }}\left(q^{-1}a\right)e^{i\varphi_{A_{\beta }}\left(q^{-1}a\right)}\}\;\\ & = & \min\{\mu_{A_{\alpha }}\left(e\right)e^{i\varphi_{A_{\beta }}\left(e\right)},\mu_{A_{\alpha }}\left(q^{-1}a\right)e^{i\varphi_{A_{\beta }}\left(q^{-1}a\right)}\}\\ & = & \mu_{A_{\alpha }}\left(q^{-1}a\right)e^{i\varphi_{A_{\beta }}\left(q^{-1}a\right)}\\ & = & \mu_{qA_{\alpha }}\left(a\right)e^{i\varphi_{qA_{\beta }}\left(a\right)}. \end{array}$$

Interchange the role of *p* and *q*, and we obtain $\mu_{qA_{\alpha }}\left(a\right)e^{i\varphi_{qA_{\beta }}\left(a\right)}\geq \mu_{pA_{\alpha }}\left(a\right)e^{i\varphi_{pA_{\beta }}\left(a\right)}$. Therefore, $\mu_{pA_{\alpha }}\left(a\right)e^{i\varphi_{pA_{\beta }}\left(a\right)}=\mu_{qA_{\alpha }}\left(a\right)e^{i\varphi_{qA_{\beta }}\left(a\right)}$.

(ii) Similarly one can prove that as part (i). □

**Theorem** **13.** *Let $A_{\left(\alpha ,\beta \right)}$ be an (α,β)-CFNSG of group G and p,q,a, and b be any elements in G. If ${pA}_{\left(\alpha ,\beta \right)}=aA_{\left(\alpha ,\beta \right)}$ and ${qA}_{\left(\alpha ,\beta \right)}=bA_{\left(\alpha ,\beta \right)}$, then ${pqA}_{\left(\alpha ,\beta \right)}=abA_{\left(\alpha ,\beta \right)}.$*

**Proof.**  Given that ${pA}_{\left(\alpha ,\beta \right)}=aA_{\left(\alpha ,\beta \right)}$ and ${qA}_{\left(\alpha ,\beta \right)}=bA_{\left(\alpha ,\beta \right)}$. This implies that ${\;p}^{-1}a,q^{-1}b\;\in \;A_{\left(\alpha ,\beta \right)}^{e}.$

Consider, ${\left(pq\right)}^{-1}\left(ab\right)=q^{-1}\left(p^{-1}a\right)b=q^{-1}\left(p^{-1}a\right)\left(qq^{-1}\right)b=\left[q^{-1}\left(p^{-1}a\right)\left(q\right)\right]\left(q^{-1}b\right)$. As $A_{\left(\alpha ,\beta \right)}^{e}$ is normal subgroup of *G*. Thus, ${\left(pq\right)}^{-1}\left(ab\right)\in A_{\left(\alpha ,\beta \right)}^{e}$. Consequently, ${pqA}_{\left(\alpha ,\beta \right)}=abA_{\left(\alpha ,\beta \right)}$.

In the following result, we establish (α,β)-complex fuzzy quotient group analog to the classical quotient group. □

**Theorem** **14.** *Let $G/A_{\left(\alpha ,\beta \right)}=\left\{{pA}_{\left(\alpha ,\beta \right)}:p\in G\right\}$ be the collection of all (α,β)-complex fuzzy cosets of (α,β)-CFNSG $A_{\left(\alpha ,\beta \right)}$ of G. Then, the binary operator 🟉 is a well-defined operation of set $G/A_{\left(\alpha ,\beta \right)}$ and is defined as $pA_{\left(\alpha ,\beta \right)}🟉qA_{\left(\alpha ,\beta \right)}=pqA_{\left(\alpha ,\beta \right)}\;\mathrm{for}\;\mathrm{all}\;$p,q∈G.*

**Proof.**  We have $pA_{\left(\alpha ,\beta \right)}=qA_{\left(\alpha ,\beta \right)}\;and\;aA_{\left(\alpha ,\beta \right)}=bA_{\left(\alpha ,\beta \right)}$, for any a,b,p,q∈G. Let g∈G be any element, then

$$\left[pA_{\left(\alpha ,\beta \right)}🟉aA_{\left(\alpha ,\beta \right)}\right]\;\left(g\right)=\left(paA_{\left(\alpha ,\beta \right)}\left(g\right)\right)=\left(g,\mu_{paA_{\alpha }}\left(g\right)e^{{i\varphi }_{paA_{\beta }}\left(g\right)}\right)$$

$$\begin{array}{ccc} \mathrm{Consider},\;\;\mu_{paA_{\alpha }}\left(g\right)e^{i\varphi_{{paA}_{\beta }}\left(g\right)} & = & \min\{\mu_{paA}\left(g\right)e^{{i\varphi }_{paA}\left(g\right)},\alpha e^{i\beta }\}\;\\ & = & \min\{\mu_{A}\left({\left(pa\right)}^{-1}g\right)e^{i\varphi_{A}\left({\left(pa\right)}^{-1}g\right)},\;\alpha e^{i\beta }\}\\ & = & \min\{\mu_{A}\left(a^{-1}\left(p^{-1}g\right)\right)e^{i\varphi_{A}\left(a^{-1}\left(p^{-1}g\right)\right)},\;\alpha e^{i\beta }\}\;\\ & = & \mu_{aA_{\alpha }}\left(p^{-1}g\right)e^{{i\varphi }_{aA_{\beta }}\left(p^{-1}g\right)}\\ & = & \mu_{bA_{\alpha }}\left(p^{-1}g\right)e^{{i\varphi }_{bA_{\beta }}\left(p^{-1}g\right)}\\ & = & \min\{\mu_{A}\left(b^{-1}\left(p^{-1}g\right)\right)e^{{I\varphi }_{A}\left(b^{-1}\left(p^{-1}g\right)\right)},\alpha e^{i\beta }\}\;\\ & = & \min\{\mu_{A}\left(p^{-1}\left(gb^{-1}\right)\right),\alpha e^{i\beta }\}\;\\ & = & \mu_{pA_{\alpha }}\left(gb^{-1}\right)e^{{i\varphi }_{pA_{\beta }}\left(gb^{-1}\right)}\\ & = & \mu_{qA_{\alpha }}\left(gb^{-1}\right)e^{{i\varphi }_{qA_{\beta }}\left(gb^{-1}\right)}\\ & = & \min\{\mu_{A}\left(q^{-1}\left({gb}^{-1}\right)\right)e^{{i\varphi }_{A}\left(q^{-1}\left({gb}^{-1}\right)\right)},\alpha e^{i\beta }\}\;\\ & = & \min\{\mu_{A}\left(q^{-1}g\right)b^{-1}e^{{i\varphi }_{A}\left(q^{-1}g\right)b^{-1}},\alpha e^{i\beta }\}\;\\ & = & \min\{\mu_{A}\left(b^{-1}q^{-1}\left(g\right)\right)e^{{I\varphi }_{A}\left(b^{-1}q^{-1}\left(g\right)\right)},\alpha e^{i\beta }\}\;\\ & = & \min\{\mu_{A}\left({\left(qb\right)}^{-1}\left(g\right)\right)e^{{I\varphi }_{A}\left({\left(qb\right)}^{-1}\left(g\right)\right)},\alpha e^{i\beta }\}\;\\ & = & \mu_{qbA_{\alpha }}\left(g\right)e^{{i\varphi }_{qbA_{\beta }}\left(g\right)}. \end{array}$$

Thus, 🟉 is well-defined operation on $G/A_{\left(\alpha ,\beta \right)}$. Note that the set $G/A_{\left(\alpha ,\beta \right)}$ fulfills the closure and associative axioms with respect to the well-defined binary operation 🟉. Further, $\mu_{A_{\alpha }}e^{i\varphi_{A_{\beta }}\;}🟉\mu_{{pA}_{\alpha }}e^{i\varphi_{pA_{\beta }}\;}$ = $\mu_{eA_{\alpha }}e^{i\varphi_{eA_{\beta }}\;}🟉\mu_{{pA}_{\alpha }}e^{i\varphi_{pA_{\beta }}\;}=\mu_{pA_{\alpha }}e^{i\varphi_{pA_{\beta }}\;}=\mu_{pA_{\alpha }}e^{i\varphi_{pA_{\beta }}\;}$ $⟹\mu_{A_{\alpha }}e^{i\varphi_{A}\;}$ is neutral element of $G/A_{\left(\alpha ,\beta \right)}$. Clearly the inverse of every element of $G/A_{\left(\alpha ,\beta \right)}\;$ exist if $\mu_{pA_{\alpha }}e^{i\varphi_{pA_{\beta }}\;}\in G/A_{\left(\alpha ,\beta \right)}$, and then there exists an element, $\mu_{p^{-1}A_{\alpha }}e^{i\varphi_{p^{-1}A_{\beta }}\;}\in G/A_{\left(\alpha ,\beta \right)}$ such that $\mu_{p^{-1}pA_{\alpha }}e^{i\varphi_{p^{-1}{pA}_{\beta }}\;}$ = $\mu_{A_{\alpha }}e^{i\varphi_{A_{\beta }}\;}.$ As a result, $G/A_{\left(\alpha ,\beta \right)}$ is a group. The group $G/A_{\left(\alpha ,\beta \right)}$ is called the quotient group of the *G* by $A_{\left(\alpha ,\beta \right)}$. □

**Lemma** **1.** *Let $h:G\to G/A_{\left(\alpha ,\beta \right)}$ be natural homomorphism from group G onto $G/A_{\left(\alpha ,\beta \right)}$ and defined by the rule, $h\left(p\right)\;=pA_{\left(\alpha ,\beta \right)}$ with the kernel h =$A_{\left(\alpha ,\beta \right)}^{e}$.*

**Proof.**  Let p,q be any elements of group *G*, and then

$$h\left(pq\right)=pqA_{\left(\alpha ,\beta \right)}=\mu_{pqA_{\alpha }}e^{i\varphi_{pqA_{\beta }}\;}=\mu_{pA_{\alpha }}e^{i\varphi_{pA_{\beta }}\;}🟉\mu_{qA_{\alpha }}e^{i\varphi_{qA_{\beta }}\;}=pA_{\left(\alpha ,\beta \right)}🟉qA_{\left(\alpha ,\beta \right)}=h\left(p\right)🟉h\left(q\right).$$

Thus, *h* is homomorphism. Further, *f* is as well

$$\begin{array}{ccc} \mathrm{Now},\;Kerh & = & \left\{p\in G:f\;\left(p\right)=eA_{\left(\alpha ,\beta \right)}\right\}\\ & = & \left\{p\in G:\;pA_{\left(\alpha ,\beta \right)}=eA_{\left(\alpha ,\beta \right)}\;\right\}\\ & = & \left\{p\in G:pe^{-1}\in A_{\left(\alpha ,\beta \right)}^{e}\right\}\\ & = & \left\{p\in G:p\in A_{\left(\alpha ,\beta \right)}^{e}\right\}\\ & = & A_{\left(\alpha ,\beta \right)}^{e}. \end{array}$$

 □

In the following result, we establish (α,β)-CFSG of the quotient group induced by the normal subgroup $A_{\alpha ,\beta }^{e}$.

**Theorem** **15.** *Let $A_{\alpha ,\beta }^{e}$ be a normal subgroup of G. If $A_{\left(\alpha ,\beta \right)}=\left\{\left(p,\mu_{A_{\alpha }}\left(p\right)e^{{i\varphi }_{A_{\beta }}\left(p\right)}\right):p\in G\right\}$ is (α,β)-CFSG, then, the (α,β)-CFS ${\bar{A}}_{\left(\alpha ,\beta \right)}=\left\{\left(pA_{\left(\alpha ,\beta \right)}^{e},{\bar{\mu }}_{A_{\alpha }}\left(pA_{\left(\alpha ,\beta \right)}^{e}\right)e^{{i\bar{\varphi }}_{A_{\beta }}\left(pA_{\left(\alpha ,\beta \right)}^{e}\right)}\right):p\in G\right\}$ of $G/A_{\left(\alpha ,\beta \right)}^{e}$ is also a (α,β)-CFSG of $G/A_{\alpha ,\beta }^{e}$, where ${\bar{\mu }}_{A_{\alpha }}\left(pA_{\left(\alpha ,\beta \right)}^{e}\right)e^{{i\bar{\varphi }}_{A_{\beta }}\left(pA_{\left(\alpha ,\beta \right)}^{e}\right)}=\max\{\mu_{A_{\alpha }}\left(pa\right)e^{i\varphi_{A_{\beta }}\left(pa\right)}:a\in A_{\left(\alpha ,\beta \right)}^{e}\}\;.$*

**Proof.**  First, we shall prove that ${\bar{\mu }}_{A_{\alpha }}\left(pA_{\left(\alpha ,\beta \right)}^{e}\right)e^{{i\bar{\varphi }}_{A_{\beta }}\left(pA_{\left(\alpha ,\beta \right)}^{e}\right)}$ is well-defined. Let ${pA}_{\alpha ,\beta }^{e}=qA_{\alpha ,\beta }^{e}$ then q=pa, for some $a\in A_{\left(\alpha ,\beta \right)}^{e}$.

$$\begin{array}{ccc} \mathrm{Now},\;\;\;\;\;\;{\bar{\mu }}_{A_{\alpha }}\left(qA_{\left(\alpha ,\beta \right)}^{e}\right)e^{{i\bar{\varphi }}_{A_{\beta }}\left(qA_{\alpha ,\beta }^{e}\right)} & = & \max\{\mu_{A_{\alpha }}\left(qb\right)e^{{i\varphi }_{A_{\beta }}\left(qb\right)}:b\in A_{\left(\alpha ,\beta \right)}^{e}\}\;\\ & = & \max\{\mu_{A_{\alpha }}\left(pab\right)e^{{i\varphi }_{A_{\beta }}\left(pab\right)}:c=ab\in A_{\left(\alpha ,\beta \right)}^{e}\}\;\\ & = & \max\{\mu_{A_{\alpha }}\left(pc\right)e^{{i\varphi }_{A_{\beta }}\left(pc\right)}:c\in A_{\left(\alpha ,\beta \right)}^{e}\}\;\\ & = & {\bar{\mu }}_{A_{\alpha }}\left(pA_{\left(\alpha ,\beta \right)}^{e}\right)\;e^{{i\bar{\varphi }}_{A_{\beta }}\left(pA_{\left(\alpha ,\beta \right)}^{e}\right)} \end{array}$$

Therefore, ${\bar{\mu }}_{A_{\alpha }}\left(pA_{\left(\alpha ,\beta \right)}^{e}\right)e^{{i\bar{\varphi }}_{A_{\beta }}\left(pA_{\left(\alpha ,\beta \right)}^{e}\right)}$ is well-defined.

$$\begin{array}{ccc} \mathrm{Consider} & & {\bar{\mu }}_{A_{\alpha }}\left\{\left(pA_{\left(\alpha ,\beta \right)}^{e}\right)\left(qA_{\left(\alpha ,\beta \right)}^{e}\right)\right\}e^{{i\bar{\varphi }}_{A_{\beta }}\left\{\left(pA_{\left(\alpha ,\beta \right)}^{e}\right)\left(qA_{\left(\alpha ,\beta \right)}^{e}\right)\right\}}\\ & = & {\bar{\mu }}_{A_{\alpha }}\left(pqA_{\left(\alpha ,\beta \right)}^{e}\right)e^{{i\bar{\varphi }}_{A_{\beta }}\left(pqA_{\left(\alpha ,\beta \right)}^{e}\right)}\\ & = & \max\{\mu_{A_{\alpha }}\left(pqa\right)e^{{i\varphi }_{A_{\beta }}\left(pqa\right)}:\;a\in A_{\left(\alpha ,\beta \right)}^{e}\}\;\\ & \geq & \max\{\min\{\mu_{A_{\alpha }}\left(pb\right)e^{{i\varphi }_{A_{\left(\alpha ,\beta \right)}}\left(pb\right)},\mu_{A_{\alpha }}\left(qc\right)e^{{i\varphi }_{A_{\beta }}\left(qc\right)}\}\;:b,c\in A_{\alpha ,\beta }^{e}\}\;\\ & = & \min\{\max\{\mu_{A_{\alpha }}\left(pb\right)e^{{i\varphi }_{A_{\beta }}\left(pb\right)}\}\;:b\in A_{\alpha ,\beta }^{e},\max\{\mu_{A_{\alpha }}\left(qc\right)e^{{i\varphi }_{A_{\beta }}\left(qc\right)}\}\;:c\in A_{\alpha ,\beta }^{e}\;\}\;\\ & = & \min\{{\bar{\mu }}_{A_{\alpha }}\left(pA_{\left(\alpha ,\beta \right)}^{e}\right)e^{{i\bar{\varphi }}_{A_{\beta }}\left(pA_{\left(\alpha ,\beta \right)}^{e}\right)},{\bar{\mu }}_{A_{\alpha }}\left(qA_{\left(\alpha ,\beta \right)}^{e}\right)e^{{i\bar{\varphi }}_{A_{\beta }}\left(qA_{\left(\alpha ,\beta \right)}^{e}\right)}\}\;. \end{array}$$

$$\begin{array}{ccc} \;\;{\bar{\mu }}_{A_{\alpha }}\left({\left(pA_{\left(\alpha ,\beta \right)}^{e}\right)}^{-1}\right)e^{{i\bar{\varphi }}_{A_{\beta }}\left({\left(pA_{\left(\alpha ,\beta \right)}^{e}\right)}^{-1}\right)} & = & {\bar{\mu }}_{A_{\alpha }}\left(p^{-1}A_{\alpha ,\beta }^{e}\right)e^{{i\bar{\varphi }}_{A_{\beta }}\left(p^{-1}A_{\alpha ,\beta }^{e}\right)}\\ & = & \max\{\mu_{A_{\alpha }}\left(p^{-1}a\right)e^{{i\varphi }_{A_{\beta }}\left(p^{-1}a\right)}:a\in A_{\alpha ,\beta }^{e}\}\;\\ & \geq & \max\{\mu_{A_{\alpha }}\left(pa\right)e^{{i\varphi }_{A_{\beta }}\left(pa\right)}:\;a\in A_{\alpha ,\beta }^{e}\}\;\\ & = & {\bar{\mu }}_{A_{\alpha }}\left(pA_{\left(\alpha ,\beta \right)}^{e}\right)e^{{i\bar{\varphi }}_{A_{\beta }}\left(pA_{\left(\alpha ,\beta \right)}^{e}\right)}. \end{array}$$

This concludes the proof. □

**Remark** **7.** *If $A_{\left(\alpha ,\beta \right)}$ is an (α,β)-CFSG of a group G, let p∈G and $\mu_{A_{\alpha }}\left(pq\right)e^{i{\varphi_{A}}_{\beta }\left(pq\right)}=\mu_{A_{\alpha }}\left(p\right)e^{i{\varphi_{A}}_{\beta }\left(p\right)}$, for all q∈G then $\mu_{A_{\alpha }}\left(p\right)e^{i{\varphi_{A}}_{\beta }\left(p\right)}=\mu_{A_{\alpha }}\left(e\right)e^{i{\varphi_{A}}_{\beta }\left(e\right)}$.*

**Definition** **15.** *Let $A_{\left(\alpha ,\beta \right)}$ be a (α,β)-complex fuzzy subgroup of finite group G. Then, the cardinality of the set $G/A_{\left(\alpha ,\beta \right)}$ of all (α,β)-complex fuzzy left cosets of G by $A_{\left(\alpha ,\beta \right)}$ is called the index of (α,β)-complex fuzzy subgroup and is denoted by $[G:A_{\left(\alpha ,\beta \right)}].$*

**Theorem** **16.**  *((α,β)-Complex Fuzzification of Lagrange’s Theorem): Let us assume that there exists a (α,β)-complex fuzzy subgroup $A_{\left(\alpha ,\beta \right)}$ of finite group G. Then, the index of (α,β)-complex fuzzy subgroup of G divides the order of G.*

**Proof.**  By Lemma 1, we have a natural homomorphism *h* from *G* to $G/A_{\left(\alpha ,\beta \right)}$.

Define a subgroup $\bar{H}=\left\{x\in G:{xA}_{\left(\alpha ,\beta \right)}={eA}_{\left(\alpha ,\beta \right)}\right\}$. By applying the Definition 13 $x\in \bar{H}$ and g∈G, we have ${xA}_{\left(\alpha ,\beta \right)}\left(g\right)={eA}_{\left(\alpha ,\beta \right)}\left(g\right)$. This implies that $A_{\left(\alpha ,\beta \right)}\left(x^{-1}g\right)=A_{\left(\alpha ,\beta \right)}\left(g\right)$, by Remark 7, which shows that $x\in A_{\left(\alpha ,\beta \right)}^{e}.$ Therefore, $\bar{H}$ is contained in $A_{\left(\alpha ,\beta \right)}^{e}.$ Now, we take any element $x\in A_{\left(\alpha ,\beta \right)}^{e}$ and using the fact $A_{\left(\alpha ,\beta \right)}^{e}$ is subgroup of *G*, we have $A_{\left(\alpha ,\beta \right)}\left(x^{-1}\right)=A_{\left(\alpha ,\beta \right)}\left(e\right).$ From Theorem 13, the elements $x^{-1},\;g\in A_{\left(\alpha ,\beta \right)}^{e},$ which means that ${xA}_{\left(\alpha ,\beta \right)}={eA}_{\left(\alpha ,\beta \right)},$ which implies that $x\in \bar{H}.$ Hence, $A_{\left(\alpha ,\beta \right)}^{e}$ is contained in $\bar{H}$. From this discussion, we can say that $\bar{H}=A_{\left(\alpha ,\beta \right)}^{e}.$

Now, we define the partition of the group *G* into the disjoint union of right cosets, and this is defined as $G=m_{1}\bar{H}$

$$\cup m_{2}\bar{H}\cup \cdots \cup m_{k}\bar{H}.\;\;\left(i\right)$$

where $m_{1}\bar{H}=\bar{H}.$ Now, we prove that, to each coset $m_{j}\bar{H}$ in relation (i), there exists an (α,β)-complex fuzzy coset $m_{j}A_{\left(\alpha ,\beta \right)}$ in $G/A_{\left(\alpha ,\beta \right)}^{e}$, and this corresponding is injective.

Consider any coset $m_{j}A_{\left(\alpha ,\beta \right)}^{e}.$ Let $x\in A_{\left(\alpha ,\beta \right)}^{e},$ then

$$\begin{array}{ccc} h\left(m_{j}x\right)=m_{j}xA_{\left(\alpha ,\beta \right)} & = & m_{j}A_{\left(\alpha ,\beta \right)}xA_{\left(\alpha ,\beta \right)}\\ & = & m_{j}A_{\left(\alpha ,\beta \right)}eA_{\left(\alpha ,\beta \right)}\\ & = & m_{j}A_{\left(\alpha ,\beta \right)}. \end{array}$$

Thus, *h* maps each element of $m_{j}A_{\left(\alpha ,\beta \right)}^{e}$ into the (α,β)-complex fuzzy coset $m_{j}A_{\left(\alpha ,\beta \right)}.$

Now, we establish a natural correspondence $\bar{h}\;$ between the set $\left\{m_{j}A_{\left(\alpha ,\beta \right)}^{e}:1\leq j\leq k\;\right\}$ and the set $G/A_{\left(\alpha ,\beta \right)}^{e}$ defined by

$$\bar{h}\left(m_{j}A_{\left(\alpha ,\beta \right)}^{e}\right)=m_{j}A_{\left(\alpha ,\beta \right)},\;1\leq j\leq k.$$

The correspondence *h* is injective.

For this, let $m_{i}A_{\left(\alpha ,\beta \right)}=m_{l}A_{\left(\alpha ,\beta \right)},$ then ${m_{l}^{-1}m}_{i}A_{\left(\alpha ,\beta \right)}=eA_{\left(\alpha ,\beta \right)}$. By using (A), we have ${m_{l}^{-1}m}_{i}\in \bar{H},$ which means that $m_{i}A_{\left(\alpha ,\beta \right)}^{e}=m_{i}A_{\left(\alpha ,\beta \right)}^{e}$, and hence $\bar{h}$ is injective. It is quite clear from the above discussion that $\left[G:\;A_{\left(\alpha ,\beta \right)}^{e}\right]\;and\;\left[G:A_{\left(\alpha ,\beta \right)}\right]$ are equal. Hence, $\left[G:\;A_{\left(\alpha ,\beta \right)}^{e}\right]$ divides O(G). □

**Example** **5.** *Consider $G=\{<u,v:u^{3}=v^{2}=e,\;uv=vu^{2}\}$ as a finite permutation group of order 6. The (α,β)-complex fuzzy subgroup $A_{\left(\alpha ,\beta \right)}$ of G corresponding to the value α=1 and β=2π is defined as*

$$A_{\left(\alpha ,\beta \right)}\left(x\right)=\;\left\{\begin{array}{c} 0.9e^{\frac{3\pi i}{2}}\;\mathrm{if}\;x=e,\\ 0.7e^{\pi i},\;\mathrm{if}\;x=u,u^{2},\\ 0.6e^{3\pi i4},\;\mathrm{otherwise}. \end{array}\right.$$

*The set of all (α,β)-complex fuzzy left cosets of G by $A_{\left(\alpha ,\beta \right)}$ is given by:*

$$G/A_{\left(\alpha ,\beta \right)}=\left\{{eA}_{\left(\alpha ,\beta \right)},\;{uA}_{\left(\alpha ,\beta \right)},\;{vA}_{\left(\alpha ,\beta \right)}\right\}.$$

*This means that $\left[G:A_{\left(\alpha ,\beta \right)}\right]=Card\left(G/A_{\left(\alpha ,\beta \right)}\right)=3.$*

## 5. Conclusions

The concept of $\left(\alpha ,\beta \right)$-CFSs is a valuable extension of classical CFSs. In this article, we defined $\left(\alpha ,\beta \right)$-CFSGs and proved fundamental algebraic attributions of these newly defined CFSGs. We presented $\left(\alpha ,\beta \right)$-complex fuzzy cosets and used these concepts to develop the $\left(\alpha ,\beta \right)$-CFNSGs. Moreover, we established an $\left(\alpha ,\beta \right)$-complex fuzzy quotient ring induced by $\left(\alpha ,\beta \right)$-CFNSG. We derived the (α,β)-complex fuzzification of Lagrange’s Theorem. In the future, we shall use the concept of the (α,β)-complex fuzzy set in algebraic structures [48,49] and decision-making problems [50]. Moreover, with the help of this newly defined complex fuzzy set, we shall propose novel (α,β)-complex fuzzy machine learning algorithms. 

## Data Availability

No real data were used to support this study. The data used in this study are hypothetical and anyone can use them by citing this article.
